# Characterizing the Assemblage of Wood-Decay Fungi in the Forests of Northwest Arkansas

**DOI:** 10.3390/jof7040309

**Published:** 2021-04-16

**Authors:** Nawaf Alshammari, Fuad Ameen, Muneera D. F. AlKahtani, Steven Stephenson

**Affiliations:** 1 Department of Biological Sciences, University of Hail, Hail 81451, Saudi Arabia; na004@uark.edu; 2 Department of Botany & Microbiology, College of Science, King Saud University, Riyadh 11451, Saudi Arabia; 3 Biology Department, College of Science, Princess Nourah Bint Abdulrahman University, Riyadh 11564, Saudi Arabia; mdfkahtani@gmail.com; 4 Department of Biological Sciences, University of Arkansas, Fayetteville, AR 72701, USA; slsteph@uark.edu

**Keywords:** basidiomycota, coarse woody debris, ITS ribosomal DNA region, Ozark Mountains

## Abstract

The study reported herein represents an effort to characterize the wood-decay fungi associated with three study areas representative of the forest ecosystems found in northwest Arkansas. In addition to specimens collected in the field, small pieces of coarse woody debris (usually dead branches) were collected from the three study areas, returned to the laboratory, and placed in plastic incubation chambers to which water was added. Fruiting bodies of fungi appearing in these chambers over a period of several months were collected and processed in the same manner as specimens associated with decaying wood in the field. The internal transcribed spacer (ITS) ribosomal DNA region was sequenced, and these sequences were blasted against the NCBI database. A total of 320 different fungal taxa were recorded, the majority of which could be identified to species. Two hundred thirteen taxa were recorded as field collections, and 68 taxa were recorded from the incubation chambers. Thirty-nine sequences could be recorded only as unidentified taxa. Collectively, the specimens of fungi collected in the forests of northwest Arkansas belong to 64 and 128 families and genera, respectively.

## 1. Introduction

Wood-decay fungi play an essential role in the decomposition of the coarse woody debris (CWD) derived from trees and other woody plants (e.g., shrubs) that make up the major component of the vegetation in forest ecosystems. The decomposition of CWD is an exceedingly critical environmental process since CWD is important in nutrient recycling, represents the primary carbon resource in ecosystems, and exerts a major influence on the development of soils [1,2,3]. The role of wood-decay fungi is of paramount importance since numerous wood-decay fungi have the capability to degrade the lignin constituent of coarse woody debris [4,5], which otherwise would accumulate over time. The association of wood-decay fungi with a given piece of CWD generally begins with the attachment of fungal mycelia and/or spores to the wood surface. The moisture retained by wood and the local temperature appear to be the most important factors for successful establishment of wood decay fungi on CWD (Pouska et al. 2016). Moreover, wood-decay fungi obtain the energy they need by absorbing molecules derived from the collapse of the cellulose component of CWD. In addition, some wood-decay fungi are also used as a source of food and a breeding place by many animals, particularly invertebrates [6].

Wood-decay fungi are common in the forests of Arkansas, but there has never been a comprehensive study to characterize the taxa present. Therefore, the study reported herein was designed both to collect wood-decay fungi in the field, as well as to isolate these organisms in the laboratory. The study was carried out in three representative study areas in northwest Arkansas—Den State Park, the Buffalo National River, and Pea Ridge National Military Park. These three study areas were selected because they occur in protected areas subjected to relatively low levels of human disturbance, are characterized by forests similar in composition and structure, were located close to the University of Arkansas (which served as the base of operations for this research project) to allow multiple visits, and could be easily accessed by means of roads or trails.

## 2. Materials and Methods

### 2.1. Collection of Specimens

The three study areas—Devil’s Den State Park (35°46′32″ N, 94°14′46″ W, elevation 454 m), Pea Ridge National Military Park (36°27′28″ N, 94°01′18″ W, elevation 484 m), and the Buffalo Nationals River (36°10′41″ N 92°25′34″ W, elevation 153 m)—are all located in the Ozark Mountains of northwest Arkansas. The study areas were located at upper slope or ridgetop positions and occurred at elevations > 457 m. Dominant vegetation consisted of oaks (*Quercus* spp.), sugar maple (*Acer saccharum*), red maple (*A. rubrum*), hickories (*Carya* spp.), and dogwood (*Cornus florida*). The vegetation was described in detail by Stephenson et al. [7]. We made collecting trips to each study area a number of times during the period of February 2018 to February 2019. On each visit, fruiting bodies of wood-decay fungi were collected using an opportunistic search method as described by Cannon and Sutton [8]. When specimens were observed in the field, they were photographed and then collected from the woody substrate with the use of a small hatchet, knife, or hand saw. Each specimen was loosely wrapped in aluminum foil and labeled with a single unique number. After a particular specimen had been collected, the diameter, length, and percent bark present were determined and recorded for the woody substrate on which it occurred.

In addition, 48 small pieces of CWD were collected from the three study areas. They were transported to the laboratory and incubated in plastic incubation chambers (30 cm × 12 cm × 6 cm) as described by Alshammari et al. [9]. A small amount of water was added to each chamber. The samples were observed for one year, and water was added to ensure that the samples remained moist. When fruiting bodies were observed, they were photographed and then removed from the CWD.

Specimens of fungi collected in the field were carried back to the laboratory, dried for approximately 24 h at a temperature of 42–55 °C on a food dehydrator, placed in plastic bags, and deposited in the herbarium of the University of Arkansas (UARK). Primary features of fruiting bodies, such as color, shape, and size, were noted and recorded before placing specimens on the food dehydrator in order to account for changes that took place upon drying. Very small fruiting bodies and some types of wood-decay fungi such as jelly fungi were placed in Eppendorf tubes and conserved directly in a refrigerator.

### 2.2. Morphological Descriptions

Historically, identification of wood-decay fungi has been based on the morphology of the fruiting body, including both macroscopic and microscopic features. The latter include such features as color, form of the fruiting body, and size (Gilbertson & Ryvarden 1986; Sotome et al. 2013). In the present study, tentative identifications were carried out using the illustrations and descriptions provided by Gilbertson and Ryvarden [10], Bessette et al. [11], Barron [12], Binion et al. [13] and Elliott and Stephenson [14].

### 2.3. DNA Extraction, PCR, and Sequencing.

For each of the morphospecies identified from the specimens collected in this project, DNA was extracted from one or more representative fruiting bodies. This was done using a package for the DNA-Promega Extraction Protocol (Promega Wizard, A1120, A1125, Madison, WI, USA). Genomic DNA amplification was carried out with the fungal-Universal primers ITS1 (5′TCCGTAGGTGAACCTGCGG-3) and ITS4 (5′-TCCTCCGCTTATTGATGC-3′) (Toju et al. 2012). PCR amplifications were performed in a thermocycler scheduled for initial denaturation at 95 °C for 5 min, followed by 35 denaturation cycles at 95 °C for 45 s, annealing at 50 °C for 45 s and extension at 72 °C for 1.5 min, and a final extension at 72 °C for 10 min. The length of the amplified products was checked on a 1% agarose gel electrophoresis using 0.5 TAE buffer, SYBR safe staining dye, and 1 kb DNA ladder (New England Biolabs, Ipswich, MI, USA). Amplicons were sent to GeneWiz (South Plainfield, NJ, USA) for Sanger sequencing. Sequences that were acquired from the latter company were edited and then blasted against the NCBI database for identification of the taxa involved. Sequences with 95 percent sequence similarity were considered to be identified to the level of species, and those with a lower percent sequence match were considered to be classified only to the level of genus. A 95 percent sequence similarity has been used in a variety of other studies, but no particular cutoff value has yet been uniformly identified for the identification of species throughout the kingdom Fungi. Index Fungorum was used as the nomenclatural standard (http:/www.indexfungorum.org/Stephenson/. accessed on 28 November 2019).

### 2.4. Calculation of Coefficient of Community Indices

The assemblages of specimens recorded from each of the three study areas were compared using a coefficient of community index [15].

Coefficient of community:

(1) $$(\mathrm{CC})=\frac{2c}{a+b}$$

where *a* is the total number of species present in the first study area (or dataset) being considered, *b* represents the total number of species in the second study area, and *c* represents the species shared in common for the two study areas being considered. The CC index ranges from 0 to 1, where 0 shows no common species shared between two study areas, and 1 indicates that all species are present in both study areas.

## 3. Results

A total of 386 specimens of macrofungi were ultimately sequenced. Out of these 386 sequences, those obtained from 66 specimens were of poor quality and thus not included in the analysis. Out of 320 good quality sequences, a total of 281 different taxa (Table S1) were identified, whereas 39 sequences could be recorded only as unidentified taxa (Table S2). The majority of these were listed as “uncultured” in the NCBI database, but presumably, in most instances, this indicates a taxon for which not enough is known for an identification to be possible. A total of 213 taxa were recorded as field collections, whereas 68 taxa were recorded from the incubation chambers. The field collections and the incubation chambers had only two species in common, *Phlebiopsis flavidoalba* and *Schizophyllum commune*.

**Figure 1:**
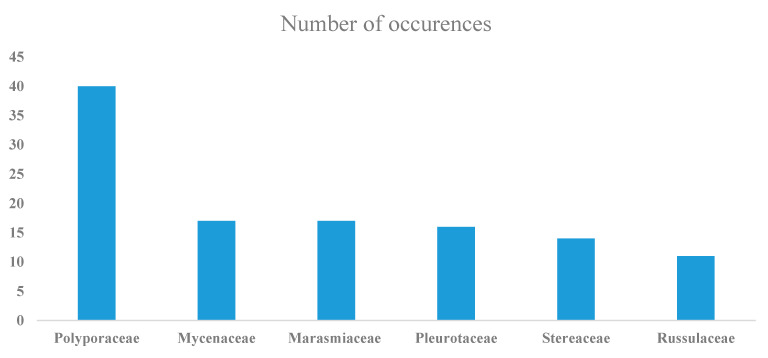
Common families of wood-decay fungi recorded in the present study.

Taxa in at least 64 different families were identified, with representatives of the Polyporaceae, Mycenaceae, Marasmiaceae, Pluteaceae, Steccherinaceae, Stereaceae, and Xylariaceae being the most common. Forty taxa were members of just the Polyporaceae. There were 128 different genera, with *Mycena* represented by the most species (17) (Figure 1). Some of the taxa identified have ecological roles not related to decomposing wood. For instance, such is the case for *Cordyceps confragosa*, which is an entomopathogenic fungus. As such, the title of this paper reflects the major emphasis of the project reported herein, which was directed towards wood-decay fungi.

The approximate volume (m^3^) was calculated for each woody substrate based on the measurements taken for length (m) and diameter (m), and the values obtained on the percentage of bark present were assigned to categories (Table 1 and Table 2). For example, the volume averages (based on > 10 occurrences) of *Merulius incarnatus, Stereum ostrea*, and *Trametes elegans* were 0.44, 0.36, and 0.25 m^3^, respectively. In contrast, *Sarcoscypha occidentalis* and *Exidia recisa* were recorded with the lowest volume averages, 6 and 22 cm^3^, respectively. Both of these species were present at a relatively early stage (percent bark <75%) of decomposition (Table 3).

**Figure 2:**
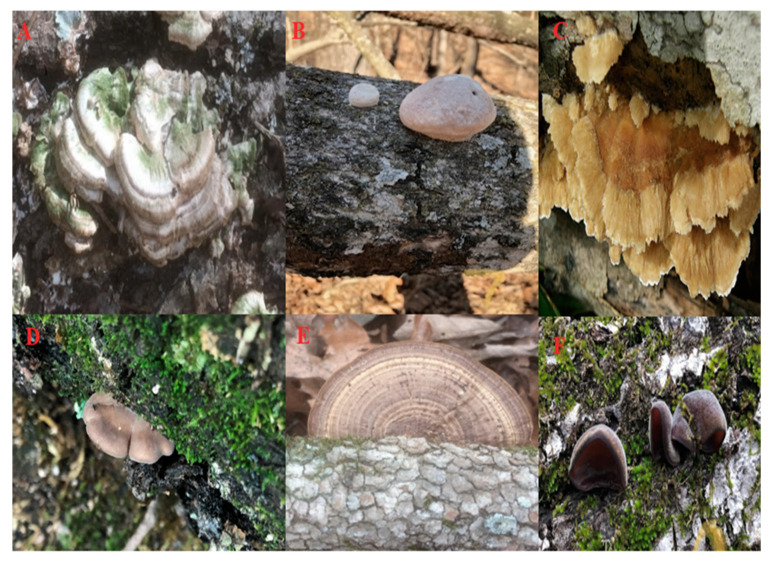
Selected species of wood-decay fungi documented from Northwest Arkansas. (**A**) *Trichaptum biforme*, (**B**) *Trametes elegans*, (**C**) *Trametopsis cervina*, (**D**) *Panellus stipticus*, (**E**) *Daedaleopsis confragosa*, and (**F**) *Auricularia americana*.

Data relating to the distribution of wood-decay fungi in the three study areas are presented Table 4. Species of wood-decay fungi such as *Trametopsis cervina*, *Panellus stypticus*, *Stereum ostrea*, and *Trichaptum biforme* occurred across all three study areas (Figure 2). However, certain species such as *Phanerochaete pseudosanguinea*, *Pluteus petasatus*, and *Hymenochaete pinnatifida* were present only in Pea Ridge Military National Park; *Daedaleopsis septentrionalis*, *Eurotium rubrum*, *Mycena haematopus*, and *Mycena inclinata* were present only in Devil’s Den State Park; and *Marasmiellus candidus*, *Rhodotus palmatus*, *Mycena zephirus*, and *Pleurotus pulmonarius* were present only in the Buffalo National River (Table 4).

The coefficient of community (CC) indices calculated for pairwise combinations of the three study areas are given in Table 5. These low values of CC among the study areas indicate that most of the species recorded were not shared in common.

## 4. Discussion

The presence of wood-decay fungi is crucial to maintain the natural processes of nutrient cycling and microbial composition in addition to maintaining the biodiversity of organisms associated with CWD or present in the adjacent soil. This becomes much more important when the regeneration of a forest largely depends upon in situ inhabitation of CWD by wood-decay fungi [2]. Therefore, the identification of wood-decay fungi can be justified for those areas where the species present are not yet known. While studies of wood-decay fungi have been carried out in various localities throughout the world (e.g., [16,17,18,19], the present study represents the most comprehensive effort to characterize the assemblage of fungi associated with the forests of northwest Arkansas. The only previous study [20] was much more limited in scope. The authors are not aware of any similar study carried out anywhere else in the Ozark Physiographic Province, which includes northern Arkansas and the southern half of Missouri and stretches westward to northern Oklahoma and southeastern Kansas.

As already noted, not all of the taxa recorded as associated with CWD are wood-decay fungi. For example, species of *Amanita* and *Russula* listed in Table S1 are ectomycorrhizal. However, the occurrence of the fruiting bodies of such fungi seemingly arising from CWD can be observed occasionally, although the fruiting body itself is ultimately connected by hyphae to a mycelium in the soil.

As the data presented in Table 1, Table 2 and Table 3 indicate, there were some patterns evident with respect to the distribution of particular taxa of fungi along the gradients represented by the diameter of the CWD, the volume of the CWD, and the stage of decay. As might be expected, both the diameter and volume of CWD were inversely correlated with the stage of decay (the more advanced the decay, the smaller the volume and diameter of the CWD). Thus, the data obtained in the present study simply verify that obtaining either measurement (with diameter being the easiest approach) will yield comparable results. It has long been known that some fungi are associated with a later stage of decay than others. As such, the data provided herein represent only a preliminary step towards developing a more complete understanding of this aspect of the ecology of wood-decay fungi in the general study area. Additional studies are clearly warranted.

It should be noted that the species of tree from which the CWD was derived could represent a factor of some importance in the distribution patterns noted above, but it was not possible to identify and assign most of the CWD to a particular tree. Much of the CWD were decorticated, and without bark being present, identification was too problematic to be attempted in many instances.

The total number of 281 taxa reported herein clearly shows that the species richness of wood-decay fungi is high in the general study area. Some of the taxa documented, including *Mycena haematopus*, *Panellus stipticus*, *Pleurotus ostreatus*, *Schizophyllum commune*, and *Stereum complicatum*, are some of the most common and widespread wood-decay fungi in the eastern United States [14]. On the other hand, an appreciable number (39) of the sequences recovered did not match anything in the GenBank database. This could mean that they are either rare (i.e., known but not yet sequenced) or possibly represent taxa new to science.

The most surprising result of this study was the fact that the assemblage of species recorded from field collections and the assemblage of species obtained in the incubation chambers were almost totally different. This was observed in our two previous studies [9,20]. In fact, the two assemblages shared only two species in common. These were *Phlebiopsis flavidoalba* and *Schizophyllum commune*. However, the differences in the two assemblages were most apparent at the level of species, and the same families and many of the same genera were represented in both assemblages. It appears likely that the difference could be attributed in part to the fact that the specimens of fungi collected in the field typically occurred on relatively large pieces of CWD, such as stumps and logs, whereas the pieces of CWD placed in the incubation chambers were mostly portions of branches no more than 3–4 cm in diameter. Closed incubation chambers have different micro-environments when compared to field conditions, and also the bagging of the substrate may have an effect. However, in spite of the variability that wood-decay fungi are known to display with respect to their biological characteristics [21], it is difficult to understand why there was such a major difference in the two assemblages of fungi. Obviously, the uniform conditions (especially in terms of temperature) that exist in incubation chambers could account for some of the difference, although it is not known why this would be the case, particularly since taxa such as *Mycena haematopus* were recorded only in incubation chambers but are not uncommon on large logs in northwestern Arkansas (Stephenson, personal observation). It is possible that the observed difference would not hold up if the number of incubation chambers was increased considerably. It should be noted that the use of incubation chambers in studies of wood-decay fungi does not appear to be a common practice. We are not aware of any other study, except our previous studies [9,22], for which incubation chambers represented a significant component of the sampling effort. However, in the present study, the incubation chambers were relatively productive, with an appreciable proportion (56%) of the chambers yielding specimens of fungi. Moreover, since the incubation chambers were monitored on a regular basis, it was possible to collect specimens of fungi when they were still in excellent condition. This is of considerable benefit in that it allows for faster detection and improves the chances of extracting high quality DNA.

In summary, the data presented herein add a considerable body of information to what is known about the mycFobiota, especially the wood-decay fungi, associated with the forests of northwest Arkansas and adjacent areas of the Ozarks. However, the relatively low numbers of taxa common to all three study areas strongly suggest that there are numerous additional taxa that are yet to be documented. As such, additional studies are clearly warranted.

## Figures and Tables

**Table 1:** Distribution of fungi in relation to the volume of the woody substrate.

	Number of Records					
Size (Volume cm^3^)	*Exidia Recisa*	*Sarcoscypha Occidentalis*	*Trametes Elegans*	*Trichaptum Biforme*	*Stereum Ostrea*	*Fuscoporia Gilva*
Large	0	0	4	3	9	2
Intermediate	0	0	6	6	14	13
Small	0	0	2	5	0	1
Very small	17	6	0	0	0	0

Note: for volume, large = 0.42 to 0.02 m^3^, intermediate = 0.021 to 0.001 m^3^, small = 1374 to 210 cm^3^, and very small = 210 to 3 cm^3^.

**Table 2:** Distribution of fungi in relation to the diameter of the woody substrate.

	Number of Records					
Size (Diameter cm)	*Exidia Recisa*	*Sarcoscypha Occidentalis*	*Trametes Elegans*	*Trichaptum Biforme*	*Stereum Ostrea*	*Fuscoporia Gilva*
Thick	1	0	2	1	3	0
Intermediate	0	0	2	4	5	5
Thin	0	0	5	4	14	7
Very thin	16	6	3	5	1	4

Note: for volume, large = 0.42 to 0.02 cm, intermediate = 0.021 to 0.001 cm, small = 1374 to 210 cm, and very small = 210 to 3 cm.

**Table 3:** Distribution of species of wood-decay fungi in relation to the stage of decay of the woody substrates from which they were collected.

Taxon	Percentage of Bark				
	75 to 100%	50 to 75%	50 to 25%	<25%	No Bark
*Stereum ostrea*	14	7	0	0	0
*Trichaptum biforme*	9	2	0	0	0
*Fuscoporia gilva*	5	6	3	0	0
*Trametes elegans*	6	2	1	0	0
*Sarcoscypha occidentalis*	6	0	0	0	0
*Exidia recisa*	9	7	0	0	0
*Phellinus stipticus*	7	0	0	0	0
*Auricularia americana*	5	0	0	0	0
*Morganella pyriformis*	5	0	0	0	0
*Deadaleopsis confragosa*	5	0	0	0	0

**Table 4:** Distribution of wood-decay fungi in the three different study areas.

All Three Study Areas	Only PRP	Only DDP	Only PFR
*Trametopsis cervina*	*Phanerochaete pseudosanguinea*	*Daedaleopsis septentrionalis*	*Marasmiellus candidus*
*Panellus stypticus*	*Pluteus petasatus*	*Eurotium rubrum*	*Rhodotus palmatus*
*Stereum ostrea*	*Hymenochaete pinnatifida*	*Hericium erinaceum*	*Mycena zephirus*
*Trichaptum biforme*	*Gloeoporus dichrous*	*Merulius incarnatus*	*Phaeomarasmius erinaceellus*
*Crucibulum laeve*	*Gymnopus erythropus*	*Mycena haematopus*	*Lactarius rubrocinctus*
*Steccherinum murashkinskyi*	*Hydnochaete tabacina*	*Mycena haematopus*	*Pleurotus pulmonarius*
*Lenzites betulinus*	*Trametes conchifer*	*Mycena inclinata*	*Pluteus glaucotinctus*
*Exidia recisa*	*Tyromyces kmetii*	*Mycena leaiana*	*Diatrype stigma*

Note: PRP: Pea Ridge Military National Park; DDP: Devil’s Den State Park; PFR: The Buffalo National River.

**Table 5:** Calculation Coefficient of community (CC) indices for the three study areas.

Study Area	Coefficient of Community (CC)		
	PRP	DDP	BFR
PRP	***	0.20	0.08
DDP	0.20	***	0.07
BFR	0.08	0.07	***

Note: PRP: Pea Ridge Military National Park; DDP: Devil’s Den State Park; PFR: the Buffalo National River. ***: No value.

## Data Availability

Accession numbers for 338 sequences of fungi generated in the present study are available at https://www.ncbi.nlm.nih.gov/nuccore/?term=Alshammari+N.+stephenson.

## Supplementary Materials

The following are available online at https://www.mdpi.com/article/10.3390/jof7040309/s1, Table S1: Taxa of wood-decay fungi recorded from northwest Arkansas. Note: %ID = percent sequence identity and SGB = sequence in GenBank. Table S2: Unidentified species of wood-decay fungi.
