# Supplementary material for: Characterizing the Assemblage of Wood-Decay Fungi in the Forests of Northwest Arkansas
Source: J Fungi (Basel). 2021 Apr 16;7(4):309. doi: 10.3390/jof7040309 (PMC8073185; doi:10.3390/jof7040309)
Supplement: Supplementary file 1 [file jof-07-00309-s001.zip › jof-1162054-supplementary.pdf]

Characterizing the assemblage of wood-decay fungi in the forests of northwest Arkansas

Nawaf I. Alshammari[1], Fuad Ameen[2*], Muneera, D.F. AlKahtani[3] and Stephenson, SL[4]

[1]Department of Biological Sciences, University of Hail, Hail, Saudi Arabia
[2]Department of Botany & Microbiology, College of Science, King Saud University, Riyadh 11451, Saudi Arabia
[3]Department of Biology, College of Science, Princess Nourah Bint Abdulrahman University, Riyadh, Kingdom of Saudi Arabia.
[4]Department of Biological Sciences, University of Arkansas, Fayetteville, Arkansas USA

Correspondence email: fuadameen@ksu.edu.sa

Supplementary tables

Table S1. Taxa of wood-decay fungi recorded from northwest Arkansas. Note: %ID = percent sequence identity and SGB = sequence in GenBank.

Taxon	Family	SGB (ITS4)	SGB (ITS1)
Acanthophysium sp. 1	Stereaceae	----------	NR_159629.1
Agaricus pinsitus Fr.	Agaricaceae	----------	MH861223.1
Agrocybe acericola (Peck) Singer	Bolbitiaceae	MN860127.1	MN860126.1
Amanita sp. 1	Amanitaceae	----------	KX348046.1
Antrodia sp. 1 (Fr.) Donk	Fomitopsidaceae	MN749971.1	MN749970.1
Antrodia serialis (Fr.) Donk	Fomitopsidaceae	----------	KC585304.1
Aspergillus ruber (Jos. König, Spieck. & W. Bremer)	Trichocomaceae	MN749936.1	MN749935.1
Auricularia Americana Parmasto & I. Parmasto	Auriculaceae	MN752647.1	MN752646.1
Blastobotrys sp. 1	Trichomonascaceae	----------	FM178345.1
Blastobotrys nivea Klopotek	Trichomonascaceae	----------	FM178345.1
Bolbitius bisporus E.F. Malysheva	Bolbitiaceae	----------	NR153611.1
Bolbitius sp. 1	Bolbitiaceae	----------	JX968249.1

Boletales sp. 1	Bolbitiaceae	MN808641.1	MN808640.1
Byssomerulius incarnates (Schwein.) Gilb.	Meruliaceae	----------	MF773635.1
Ceraceomyces. sp. 1	Amylocorticiaceae	----------	MH863804.1
Ceriporiopsis sp. 1	Phanerochaetaceae	----------	NR_154636.1
Cerrena unicolor (Bull.) Murrill	Cerrenaceae	MT050490.1	--------
Cladosporium cladosporioides (Fresen.) G.A. de Vries	Davidiellaceae	----------	MF476049.1
Clitocybe subditopoda Peck	Tricholomataceae	----------	KM453734.1
Clitopilus hobsonii (Berk.) P.D. Orton	Entolomataceae	MT032481.1	--------
Clitopilus sp. 1	Entolomataceae	MN826227.1	MN826226.1
Clitopilus sp. 2	Entolomataceae	MN826225.1	MN826224.1
Clitopilus sp. 3	Entolomataceae	MN826075.1	MN826074.1

Table S1. Continued.

Taxon	Family	SGB (ITS4)	SGB (ITS1)
Coprinellus radians (Fr.) Vilgalys, Hopple & Jacq. Johnson	Psathyrellaceae	MN753980.1	MN753979.1
Coprinellus sp. 1	Psathyrellaceae	----------	KX611630.1
Coprinus alnivorus Bogart	Coprinaceae	----------	MK169326.1
Cordyceps cateniannulata (Z.Q. Liang) Kepler, B. Shrestha & Spatafora	Cordycipitaceae	MN860177.1	MN860176.1
Cordyceps confragosa (Mains) G.H. Sung, J.M. Sung, Hywel-Jones & Spatafora	Clavicipitaceae	----------	KT372853.1
Crepidotus applanatus (Pers.) P. Kumm.	Cortinariaceae	MN826231.1	MN826230.1
Crepidotus sp. 1	Crepidotaceae	----------	MF161223.1
Crinipellis sp. 2	Crepidotaceae	MN977192.1	--------
Crucibulum leave (Huds.) Kambly	Nidulariaceae	MN749576.1	MN749575.1
Cryptococcus yokohamensis Alshahni, Satoh & Makimura	Tremellaceae	----------	HM222928.1

Cyathus annulatus H.J. Brodie	Agaricaceae	MT032353.1	--------
Cyathus renweii T.X. Zhou & R.L. Zhao	Agaricaceae	----------	NR_119589.1
Daedaleopsis confragosa (Bolton) J. Schröt.	Polyporaceae	MN749637.1	MN749636.1
Daedaleopsis septentrionalis (P. Karst.) Niemela	Polyporaceae	MN749933.1	MN749932.1
Daedaleopsis sinensis (Lloyd) Y.C. Dai	Polyporaceae	----------	FJ627256.1
Daedaleopsis tricolor (Bull.) Bondartsev & Singer	Polyporaceae	----------	KY235366.1
Daldinia childiae J.D. Rogers & Y.M. Ju	Hypoxylaceae	MN860149.1	MN860148.1
Diatrype stigma (Hoffm.) Fr.	Diatrypaceae	MN809140.1	MN809139.1

Table S1. Continued.

**Taxon**	**Family**	**SGB (ITS4)**	**SGB (ITS1)**
Donkia pulcherrima (Berk. & M.A. Curtis) Pilát	Phanerochaetaceae	MN860136.1	--------
Ductifera pululahuana (Pat.) Donk	Exidiaceae	MN860170.1	MN860169.1
Eichleriella sp. 1	Exidiaceae	----------	MH349728.1
Entoloma platyphylloides (Romagn.) Largent	Entolomataceae	----------	JF908003.1
Eurotium rubrum Jos. König, Spieck. & W. Bremer	Trichocomaceae	----------	EU001331.1
Eurotium tuberculatum Z.T. Qi & Z.M. Sun	Aspergillaceae,	----------	HE615134.1
Exidia recisa (Ditmar) Fr.	Auriculariaceae	----------	LC098751.1
Exidia sp. 1	Auriculariaceae	MN752654.1	MN752653.1
Exidia sp. 2	Auriculariaceae	----------	MF161299.1
Fuscoporia gilva (Schwein.) T. Wagner & M. Fisch.	Hymenochaetaceae	MN781978.1	MN781977.1

Galerina triscopa (Fr.) Kühner	Sarcosomataceae	MT050467.1	---------
Galiella rufa (Schwein.) Nannf. & Korf	Sarcosomataceae	MN826132.1	MN826131.1
Ganoderma sp. 1	Ganodermataceae	MN964002.1	MN964001.1
Ganoderma sp. 2	Ganodermataceae	----------	AF255100.1
Ganoderma sp. 3	Ganodermataceae	MN749648.1	MN749647.1
Gloeoporus dichrous (Fr.) Bres.	Meruliaceae	MN749626.1	MN749625.1
Grammothele sp. 1	Polyporaceae	----------	NR_158484.1
Gymnopilus decipiens (Sacc.) P.D. Orton	Cortinariaceae	MN964270.1	MN964269.1
Gymnopus biformis (Peck) Halling	Omphalotaceae	----------	KJ416250.1
Gymnopus dichrous (Berk. & M.A. Curtis) Halling	Marasmiaceae	MN864292.1	MN864291.1
Gymnopus dryophilus (Bull.) Murrill	Omphalotaceae	----------	DQ449974.1

Table S1. Continued.

Taxon	Family	SGB (ITS4)	SGB (ITS1)
Gymnopus earleae Murrill	Marasmiaceae	----------	DQ449994.1
Gymnopus erythropus (Pers.) Antonín, Halling & Noordel.	Omphalotaceae	----------	KY950460.1
Gymnopus foliiphilus R.H. Petersen	Omphalotaceae	----------	KY026721.1
Gymnopus gibbosus (Corner) A.W. Wilson, Desjardin & E. Horak	Omphalotaceae	----------	KP012713.1
Gymnopus junquilleus R.H. Petersen & J.L. Mata	Omphalotaceae	----------	KF007938
Gymnopus luxurians (Peck) Murrill	Marasmiaceae	MN860558.1	=====
Gymnopus semihirtipes (Peck) Halling	Marasmiaceae	MT027500.1	---------
Gymnopus sp. 1	Omphalotaceae	----------	MK532854.1
Gymnopus spongiosus (Berk. & M.A. Curtis) Halling	Marasmiaceae	----------	KY026687.1

Gymnopus subnudus (Ellis ex Peck) Halling	Marasmiaceae	MT027360.1	---------
*Heimiomyces neovelutipe*s (Hongo) E. Horak	Mycenaceae	---------	KM975407.1
Hericium erinaceus (Bull.) Pers.	Hericiaceae	MN752252.1	MN752251.1
Heterobasidion araucariae P.K. Buchanan	Bondarzewiaceae	---------	MH268104.1
Hohenbuehelia angustata (Berk.) Singer	Pleurotaceae	MN808821.1	MN808820.1
Hohenbuehelia petaloides (Bull.) Schulzer	Pleurotaceae	---------	GQ142023.1
Hydnochaete tabacina (Berk. & M.A. Curtis ex Fr.) Ryvarden	Hymenochaetaceae	MN749624.1	MN749623.1
Hymenochaete pinnatifida Burt	Hymenochaetaceae	MN749653.1	MN749652.1
Hymenochaete rubiginosa (Dicks.) Lév.	Hymenochaetaceae	MT032360.1	--------
Hymenochaete sp. 1	Hymenochaetaceae	---------	MH211729.1

Table S1. Continued.

Taxon	Family	SGB (ITS4)	SGB (ITS1)
Hyphodermella rosae (Bres.) Nakasone	Phanerochaetaceae	MN749631.1	MN749630.1
Hyphodontia tropica Sheng H. Wu	Hymenochaetaceae	MN752433.1	MN752432.1
Hypholoma sp. 1	Strophariacea	---------	KY950514.1
Hypocrea peltata Berk.	Hypocreaceae	---------	AB742524.1
Hypomyces microspermus Rogerson & Samuels	Hypocreaceae	MN872844.1	MN872843.1
Hypoxylon crocopeplum Berk. & M.A. Curtis	Xylariaceae	MN964272.1	MN964271.1
Infundibulicybe gibba (Pers.) Harmaja	Tricholomataceae	---------	MG663274.1
Inocybe sp. 1	Inocybaceae	MN964335.1	MN964334.1
Inocybe subradiata Murrill	Inocybaceae	---------	MF992157.1
Irpex lacteus (Fr.) Fr.	Steccherinaceae	---------	KT272411.1
Lachnum virgineum (Batsch) P. Karst.	Hyaloscyphaceae	---------	AB481268.1

Lactarius purpureocastaneus X.H. Wang	Russulaceae	----------	MF508965.1
Lactarius rubrocinctus Fr.	Russulaceae	MN872840.1	MN871815.1
Lactarius subserifluus Longyear	Russulaceae	----------	EU819486.1
Leiotrametes lactinea (Berk.) Welti & Courtec.	Polyporaceae	MN860138.1	MN860137.1
Lentinellus brunnescens Lj.N. Vassiljeva	Russulaceae	MN860129.1	MN860128.1
Lentinellus castoreus (Fr.) Kühner & Maire	Auriscalpiaceae	MN809125.1	MN809124.1
Lentinellus sp. 1	Auriscalpiaceae	----------	AY513169.1
Lentinellus sp. 2	Auriscalpiaceae	----------	MH211871.1
Lentinus levis (Berk. & M.A. Curtis) Murrill	Polyporaceae	MN809143.1	MN809142.1
Lenzites betulinus (L.) Fr.	Polyporaceae	MN749633.1	MN749632.1
Lenzites sp.1	Polyporaceae	----------	MK575249.1

Table S1. Continued.

**Taxon**	**Family**	**SGB (ITS4)**	**SGB (ITS1)**
Lepiota phaeosticta Morgan	Agaricaceae	MN826136.1	MN826135.1
Lepiota sp. 1	Agaricaceae	----------	MH212044.1
Leucoagaricus americanus (Peck) Vellinga	Agaricaceae	MN808631.1	MN808630.1
Lycoperdon pyriforme Schaeff.	Agaricaceae	----------	MF161171.1
Marasmiellus candidus (Fr.) Singer	Marasmiaceae	MN977325.1	MN860121.1
Marasmiellus juniperinus Murrill	Marasmiaceae	----------	NR_119582.1
Marasmiellus rhizomorphigenus Antonín, Ryoo & H.D. Shin	Omphalotaceae	----------	LT716051.1
Marasmiellus sp. 1	Meruliaceae	----------	MH856003.1
Marasmius sp. 2	Meruliaceae	MN872842.1	MN872841.1
Marasmius sp. 3	Meruliaceae	MN871792.1	MN871791.1

Marasmius cohaerens (Pers.) Cooke & Quél.	Marasmiaceae	----------	KF774176.1
Marasmius graminicola Speg.	Marasmiaceae	----------	FJ917619.1
Marasmius oreades (Bolton) Fr.	Marasmiaceae	----------	JN943604.1
Marasmius pulcherripes Peck	Marasmiaceae	MT032485.1	-------
Marasmius rotula (Scop.) Fr.	Marasmiaceae	----------	KC176355.1
Marasmius sp. 1	Marasmiaceae	----------	AY216476.1
Marasmius sp. 2	Marasmiaceae	----------	DQ182506.1
Mariannaea sp. 1	Nectriaceae	----------	NR_148078.1
Merulius incarnates Schwein.	Meruliaceae	MN782000.1	MN781999.1
Microstoma floccosum (Sacc.) Raitv.	Sarcoscyphaceae	----------	AF026309.1
Mollisia incrustata (Ellis) Sacc.	Dermateaceae	MN860141.1	MN860140.1
Morganella pyriformis (Schaeff.) Kreisel & D. Krüger.	Agaricaceae	----------	LT635437.1
Morganella subincarnata (Peck) Kreisel & Dring	Agaricaceae	MN964264.1	-------

Table S1. Continued.

Taxon	Family	SGB (ITS4)	SGB (ITS1)
Mortierella sp. 1	Mortierellaceae	----------	MF423523.1
Mycena acicula (Schaeff.) P. Kumm.	Mycenaceae	MN781990.1	MN781989.1
Mycena amicta (Fr.) Quél.	Mycenaceae	MT027358.1	-------
Mycena aurantiomarginata (Fr.) Quél.	Mycenaceae	----------	JF908479.1
Mycena haematopus (Pers.) P. Kumm	Mycenaceae	MN749929.1	-------
Mycena inclinata (Fr.) Quél	Mycenaceae	MT027356.1	-------
Mycena leaiana (Berk.) Sacc.	Mycenaceae	MN749928.1	MN749927.1
Mycena niveipes (Murrill) Murrill	Mycenaceae	----------	MG748570.1
Mycena polygramma (Bull.) Gray	Mycenaceae	----------	FJ917615.1
Mycena sp. 1	Mycenaceae	MN781994.1	MN781993.1
Mycena sp. 2	Mycenaceae	----------	KJ206984.1
Mycena sp. 3	Mycenaceae	----------	MK290378.1

Mycena thymicola Velen.	Mycenaceae	----------	JF908483.1
Mycena zephirus (Fr.) P. Kumm.	Mycenaceae	MN781987.1	MN781986.1
Mycetinis opacus (Berk. & M.A. Curtis) A.W. Wilson & Desjardin	Marasmiaceae	----------	KY696767.1
Mycorrhaphium adustum (Schwein.) Maas Geest.	Steccherinaceae	----------	JN710573.1
Nectria mariannaeae Samuels & Seifert	Nectriaceae	----------	GU586835.1
Neofavolus alveolaris (DC.) Sotome & T. Hatt.	Polyporaceae	----------	KP283508.1
Neofavolus sp. 1	Polyporaceae	MN860132.1	MN860131.1
Neofavolus sp. 2	Polyporaceae	MN860125.1	MN860124.1
Neofavolus sp. 3	Polyporaceae	MN752652.1	MN752651.1
Neofavolus sp. 4	Polyporaceae	MN749925.1	MN749924.1
Neofavolus sp. 5	Polyporaceae	MN749923.1	MN749922.1
Nigroporus vinosus (Berk.) Murrill	Steccherinaceae	----------	JX109857.1

Table S1. Continued.

**Taxon**	**Family**	**SGB (ITS4)**	**SGB (ITS1)**
Panellus sp. 1	Mycenaceae	----------	MK399806.1
Panellus stipticus (Bull.) P. Karst.	Mycenaceae	MN749586.1	MN749585.1
Panus conchatus (Bull.) Fr.	Panaceae	MT032480.1	-------
Panus lecomtei (Fr.) Corner	Polyporaceae	----------	KP135329.1
Panus neostrigosus Drechsler-Santos & Wartchow	Polyporaceae	----------	KU761235.1
Panus rudis Fr.	Polyporaceae	----------	KU863048.1
Perenniporia ohiensis (Berk.) Ryvarden	Polyporaceae	----------	FJ411096.1
Pezizomycetes sp. 1	Pezizomycetes	MN964250.1	MN964249.1
Pezizomycetes sp. 2	Pezizomycetes	----------	JQ761310.1
Phaeomarasmius erinaceellus (Peck) Singer	Inocybaceae	MN795482.1	MN795481.1

Phanerochaete pseudosanguinea Floudas &Hibbett	Phanerochaetaceae	MN749651.1	MN749650.1
Phanerochaete sanguinea (Fr.) Pouzar	Phanerochaetaceae	MT027238.1	-------
Phanerochaete sordida (P. Karst.) J. Erikss. & Ryvarden	Phanerochaetaceae	----------	MF476014.1
Phanerochaete sp. 1	Phanerochaetaceae	----------	MF399407.1
*Phellinus robiniae (*Murrill) A. Ames	Hymenochaetaceae	----------	KX065962.1
Phlebia tremellosa (Schrad.) Nakasone & Burds.	Meruliaceae	----------	KJ668481.1
Phlebiopsis flavidoalba (Cooke) Hjortstam	Phanerochaetaceae	----------	KX065956.1
Pholiota multifolia (Peck) A.H. Sm. & Hesler	Strophariaceae	MT027490.1	-------
Pholiota polychroa (Berk.) A.H. Sm. & H.J. Brodie	Strophariaceae	MN749584.1	MN749583.1
Pholiotina aeruginosa (Romagn.) M.M. Moser	Bolbitiaceae	MT028143.1	-------

Table S1. Continued.

**Taxon**	**Family**	**SGB (ITS4)**	**SGB (ITS1)**
Physalacria sp. 1	Physalacriaceae	----------	DQ097367.1
Physisporinus pouzarii (Vampola & Vlasák) F. Wu, Jia J. Chen & Y.C. Dai	Meripilaceae	MN826134.1	MN826133.1
Physisporinus vitreus (Pers.) P. Karst.	Meripilaceae	----------	KF800254.1
Pleurotus dryinus (Pers.) P. Kumm.	Pleurotaceae	----------	MH211881.1
Pleurotus floridanus Singer	Bolbitiaceae	----------	MG819742.1
Pleurotus ostreatus (Jacq. ex Fr.) P. Kumm.	Pleurotaceae	----------	MH395969.1
Pleurotus pulmonarius (Fr.) Quél	Pleurotaceae	MN808603.1	MN808602.1
Pleurotus sapidus Sacc.	Pleurotaceae	----------	KY962449.1
Pleurotus sp. 1	Pleurotaceae	MT027258.1	-------
Pleurotus sp. 2	Pleurotaceae	MN860173.1	MN860172.1

Pleurotus sp. 3	Pleurotaceae	MN860144.1	MN860143.1
Pleurotus sp. 4	Pleurotaceae	MN809106.1	MN809105.1
cervinus (Schaeff.) P. Kumm.	Pleurotaceae	MN749645.1	MN749644.1
Pluteus petasatus (Fr.) Gillet	Pleurotaceae	MN749639.1	MN749638.1
Pluteus romellii (Britzelm.) Sacc.	Pleurotaceae	MN752313.1	MN752312.1
Pluteus cervinus (Schaeff.) P. Kumm.	Pleurotaceae	----------	KF306014.1
Pluteus chrysophlebius (Berk. & M.A. Curtis) Sacc.	Pleurotaceae	MN860123.1	MN860122.1
Pluteus glaucotinctus E. Horak	Pluteaceae	----------	MH211860.1
Pluteus hispidulus (Fr.) Gillet	Pluteaceae	----------	KM983696.1
Pluteus hongoi Singer	Pleurotaceae	MN752233.1	MN752232.1
Pluteus longistriatus (Peck) Peck	Pluteaceae	MT032365.1	-------
Pluteus pellitus (Pers.) P. Kumm.	Pluteaceae	----------	MH211659.1
Pluteus petasatus (Fr.) Gillet	Pluteaceae	----------	KJ009707.1
Pluteus romellii (Britzelm.) Sacc.	Pluteaceae	----------	KM983699.1
Pluteus sp. 1	Pluteaceae	----------	KM983694.1

Table S1. Continued.

**Taxon**	**Family**	**SGB (ITS4)**	**SGB (ITS1)**
Pluteus thomsonii sensu Singer	Pluteaceae	----------	KX216328.1
Polyporales sp.	Polyporaceae	MN809134.1	MN809133.1
Polyporus alveolaris (DC.) Bondartsev & Singer	Polyporaceae	----------	KJ140677.1
Polyporus sp. 1	Polyporaceae	----------	AB746931.1
Polyporus sp. 2	Polyporaceae	----------	KU324794.1
Polyporus tuberaster (Jacq. ex Pers.) Fr.	Polyporaceae	MT032482.1	--------
Polyporus varius (Pers.) Fr.	Polyporaceae	MT050461.1	MT050460.1
Psathyrella sp. 1	Psathyrellaceae	----------	KC992949.1
Pseudochaete tabacina (Sowerby) T. Wagner & M. Fisch.	Hymenochaetaceae	----------	KJ140591.1

Resupinatus alboniger (Pat.) Singer	Pleurotaceae	MN752401.1	MN752400.1
Resupinatus applicatus (Batsch) Gray	Tricholomataceae	----------	KU355368.1
Rhizomarasmius pyrrocephalus (Berk.) R.H. Petersen	Psathyrellaceae	MN752228.1	MN752227.1
Rhizopus oryzae Went & Prins. Geerl.	Mucoraceae	----------	FJ478087.1
Rhodocollybia badiialba (Murrill) Lennox	Omphalotaceae	MN826119.1	MN826118.1
Rhodotus sp. 1	Physalacriaceae	----------	MG748585.1
Rigidoporus pouzarii Vampola & Vlasák	Meripilaceae	----------	JQ733558.1
Rigidoporus sp. 1	Meripilaceae	----------	MG845229.1
Russula pectinatoides Peck	Russulaceae	----------	MH211829.1
Sarcoscypha korfiana F.A. Harr.	Sarcoscyphaceae	----------	AF026308.2
Sarcoscypha occidentalis (Schwein.) Sacc.	Sarcoscyphaceae	MN808749.1	MN808748.1
Schizophyllum commune Fr.	Schizophyllaceae	MN781968.1	MN781967.1
Schizophyllum radiatum Fr.	Schizophyllaceae	----------	LT217545.1

Table S1. Continued.

**Taxon**	**Family**	**SGB (ITS4)**	**SGB (ITS1)**
Schizopora ovispora (Corner) Hjortstam & Ryvarden	Schizoporaceae	----------	KX857803.1
Scutellinia crinite (Bull.) Lambotte.	Pyronemataceae	MN752431.1	MN752430.1
Scutellinia sp. 1	Pyronemataceae	----------	MF230412.1
Simocybe serrulata (Murrill) Singer	Inocybaceae	MN872846.1	MN872845.1
Simocybe sp. 1	Inocybaceae	MN860159.1	MN860158.1
Simocybe sp. 2	Inocybaceae	MN809131.1	MN809130.1
Simplicillium aogashimaense Nonaka, Kaifuchi & Masuma	Cordycipitaceae	MT027492.1	MT027491.1
Simplicillium lanosoniveum (J.F.H. Beyma) Zare & W. Gams	Cordycipitaceae	----------	AB758126.1

Skeletocutis nivea (Jungh.) Jean Keller	Polyporaceae	MN781970.1	MN781969.1
Skeletocutis semipileata (Peck) Miettinen & A. Korhonen	Polyporaceae	MN964075.1	MN964074.1
Spongipellis pachyodon (Pers.) Kotl. & Pouzar	Hapalopilaceae	MT027357.1	---------
Steccherinum bourdotii Saliba & A. David	Steccherinaceae	MN749594.1	MN749593.1
Steccherinum laeticolor (Berk. & M.A. Curtis) Banker	Steccherinaceae	MT027254.1	---------
*Steccherinum murashkinskyi (*Burt) Maas Geest.	Steccherinaceae	----------	FJ798705.1
Steccherinum undigerum (Berk. & M.A. Curtis) Westphalen & Tomšovský	Steccherinaceae	MN781992.1	MN781991.1
Stereum complicatum (Fr.) Fr.	Stereaceae	MN749663.1	MN749662.1
Stereum hirsutum (Willd.) Pers.	Stereaceae	----------	MH211730.1
Stereum ostrea (Blume & T. Nees) Fr.	Stereaceae	MN749635.1	MN749634.1
Stereum sanguinolentum (Alb. & Schwein.) Fr.	Stereaceae	MN864252.1	MN864251.1

Table S1. Continued.
.

**Taxon**	**Family**	**SGB (ITS4)**	**SGB (ITS1)**
Stereum sp. 1	Stereaceae	MN965750.1	MN965749.1
Stereum sp. 2	Stereaceae	MN864258.1	MN864257.1
Stereum sp. 3	Stereaceae	MN752263.1	MN752262.1
Stereum sp. 4	Stereaceae	MN749665.1	MN749664.1
Stereum sp. 5	Stereaceae	----------	KJ831876.1
Stereum sp. 6	Stereaceae	----------	KJ831881.1
Stereum sp. 7	Stereaceae	----------	MH268105.1
Strobilomyces sp.	Boletaceae	----------	MK733920.1
Tetrapyrgos nigripes (Fr.) E. Horak	Marasmiaceae	----------	DQ449942.1
Theleporus sp. 1	Grammotheleaceae	----------	NR_119985.1

Tomentella sp. 1	Thelephoraceae	----------	EU625920.1
Trametes conchifer (Schwein.) Pilát	Polyporaceae	MN749629.1	MN749628.1
Trametes cubensis (Mont.) Sacc.	Polyporaceae	MN860166.1	MN860165.1
Trametes elegans (Spreng.) Fr.	Polyporaceae	MN749588.1	MN749587.1
Trametes hirsute (Wulfen) Lloyd	Polyporaceae	----------	GQ280373.1
Trametes sp. 1	Polyporaceae	MN964006.1	MN964005.1
Trametes sp. 2	Polyporaceae	MN752650.1	MN752649.1
Trametes versicolor (L.) Lloyd	Polyporaceae	MN749590.1	MN749589.1
Trametes villosa (Sw.) Kreisel	Polyporaceae	MN781670.1	MN781669.1
Trametopsis cervina (Schwein.) Tomšovský	Polyporaceae	MN749931.1	MN749930.1
Trametopsis sp. 1	Hapalopilaceae	----------	MG663240.1
Tremella yokohamensis (Alshahni, Satoh & Makimura) Yurkov	Tremellaceae	MN752643.1	MN752642.1
Trichaptum biforme (Fr.) Ryvarden	Polyporaceae (?)	MN964276.1	MN964275.1
Trichaptum fuscoviolaceum (Ehrenb.) Ryvarden	Polyporaceae	----------	MF381026.1

Table S1. Continued.

**Taxon**	**Family**	**SGB (ITS4)**	**SGB (ITS1)**
Trichoderma atroviride P. Karst.	Hypocreaceae	MT027497.1	-------
Trichoderma gamsii Samuels & Druzhin.	Hypocreaceae	----------	KX009501.1
Trichoderma sp. 1	Hypocreaceae	MT027495.1	-------
Trichoderma sp. 2	Hypocreaceae	----------	AB872440.1
Trichoderma viride Pers.	Hypocreaceae	----------	KM458804.1
Trogia furcata Corner	Marasmiaceae	----------	MF100962.1
Truncospora ohiensis (Berk.) Pilát	Polyporaceae	MT027494.1	MT027493.1
Truncospora sp. 1	Polyporaceae	----------	KP768411.1
Tylopilus felleus (Bull.) P. Karst.	Boletaceae	MT032364.1	--------

Tylopilus rubrobrunneus Mazzer & A.H. Sm.	Boletaceae	----------	GQ166869.1
Tyromyces galactinus (Berk.) Bondartsev	Polyporaceae	MN872818.1	MN872817.1
Tyromyces kmetii (Bres.) Bondartsev & Singer	Polyporaceae	MN749592.1	MN749591.1
Urnula craterium (Schwein.) Fr.	Sarcosomataceae	MN781996.1	MN781995.1
Xeromphalina kauffmanii A.H. Sm.	Mycenaceae	----------	MG663296.1
Xylaria cornu-damae (Schwein.) Berk.	Xylariaceae	MT027514.1	--------
Xylaria heliscus (Mont.) J.D. Rogers & Y.M. Ju	Xylariaceae	MT027515.1	--------
Xylaria hypoxylon (L.) Grev.	Xylariaceae	----------	U47841
Xylaria sp. 1	Xylariaceae	MT028182.1	--------
Xylaria sp. 2	Xylariaceae	----------	KU683962.1
Xylobolus subpileatus (Berk. & M.A. Curtis) Boidin	Stereaceae	MN750025.1	MN750024.1
Xylodon subflaviporus C.C. Chen & Sheng H. Wu	Stereaceae	MN749657.1	MN749656.1

Table. S2. Unidentified species of wood-decay fungi.

**Taxon**	**SGB (ITS4)**	**SGB (ITS1)**
Unidentified fungus	MN872814.1	MN872833.1
Unidentified fungus	MN964278.1	MN964277.1
Unidentified fungus	MN872837.1	MN872836.1
Unidentified fungus	MN872835.1	MN872834.1
Unidentified fungus	MN872830.1	MN872829.1
Unidentified fungus	MN872824.1	MN872823.1
Unidentified fungus	MN872826.1	----------
Unidentified fungus	MN872822.1	MN872821.1
Unidentified fungus	MN872820.1	MN872819.1
Unidentified fungus	MN872816.1	MN872815.1

Unidentified fungus	MN872811.1	MN872810.1
Unidentified fungus	MN872809.1	MN872808.1
Unidentified fungus	MN872807.1	MN872806.1
Unidentified fungus	MN831409.1	MN831408.1
Unidentified fungus	MN831407.1	MN831406.1
Unidentified fungus	MN831389.1	MN831388.1
Unidentified fungus	MN831401.1	MN831400.1
Unidentified fungus	MN831385.1	-----------
Unidentified fungus	MN831387.1	MN831384.1
Unidentified fungus	------------	MN831386.1
Unidentified fungus	MN831405.1	MN831404.1
Unidentified fungus	MN831381.1	MN831380.1
Unidentified fungus	MN831391.1	MN831390.1
Unidentified fungus	MN831393.1	MN831392.1
Unidentified fungus	MN831399.1	------------
Unidentified fungus	MN831395.1	MN831398.1
Unidentified fungus	------------	MN831394.1
Unidentified fungus	MN831397.1	MN831396.1

Table. S2 Continued.

**Taxon**	**SGB (ITS4)**	**SGB (ITS1)**
Unidentified fungus	MN831383.1	--------------
Unidentified fungus	MN831403.1	MN831382.1
Unidentified fungus	---------	MN831402.1
Unidentified fungus	MN781976.1	MN781975.1
Unidentified fungus	----------	MN872825.1
Unidentified fungus	----------	MN860557.1
fungal sp. 1	MT027496.1	-------------
fungal sp. 2	MN860156.1	MN860155.1
fungal sp. 3	MN826229.1	MN826228.1

fungal sp. 4	MN749668.1	MN749667.1
